# The Risks of Sleeping “Too Much”. Survey of a National Representative Sample of 24671 Adults (INPES Health Barometer)

**DOI:** 10.1371/journal.pone.0106950

**Published:** 2014-09-16

**Authors:** Damien Léger, François Beck, Jean-Baptiste Richard, Fabien Sauvet, Brice Faraut

**Affiliations:** 1 Centre du Sommeil et de la Vigilance, Hôtel Dieu, APHP, Université Paris Descartes, Sorbonne Paris Cité, Paris, France; 2 VIFASOM, équipe d'accueil Vigilance Fatigue et Sommeil, Université Paris Descartes, Sorbonne Paris Cité, Paris, France; 3 Equipe Cesames (centre de recherche médecine, sciences, santé, santé mentale, société, Université Paris Descartes/CNRS UMR 8211/Inserm U988/EHESS), Cermes 3, Direction des Affaires Scientifiques INPES, Saint Denis, France; 4 Institut de recherche biomédicale des armées (IRBA), Brétigny-sur-Orge, France; University of Oxford, United Kingdom

## Abstract

**Background:**

A significant U-shaped association between sleep duration and several morbidity (obesity, diabetes or cardiovascular disease) and mortality risks has been regularly reported. However, although the physiological pathways and risks associated with “too short sleep” (<5 hours/day) have been well demonstrated, little is known about “too much sleeping”.

**Purpose:**

To explore socio-demographic characteristics and comorbidities of “long sleepers” (over 10 hours/day) from a nationally representative sample of adults.

**Methods:**

A cross-sectional nationally representative sample of 24,671 subjects from 15 to 85-year-old. An estimated total sleep time (TST) on non-leisure days was calculated based on a specifically designed sleep log which allows to distinguish “long sleepers” from “short sleepers” (<5 hours/day). Insomnia was assessed according to the International classification of sleep disorders (ICSD-2).

**Results:**

The average TST was 7 hours and 13 minutes (+/− 17 minutes). Six hundred and twelve subjects were “long sleepers” (2.7%) and 1969 “short sleepers” (7.5%). Compared to the whole group, “long sleepers” were more often female, younger (15–25 year-old) or older (above 65 year-old), with no academic degree, mostly clerks and blue collar workers. “Long sleepers” were significantly more likely to have psychiatric diseases and a greater body mass index (BMI). However, long sleep was not significantly associated with the presence of any other chronic medical disease assessed. Conversely, short sleep duration was significantly associated with almost all the other chronic diseases assessed.

**Conclusions:**

In the general population, sleeping too much was associated with psychiatric diseases and higher BMI, but not with other chronic medical diseases.

## Introduction

It is generally recommended as one of the major rules for good health in children, but also in adults, to have a sufficient amount of sleep everyday [1]–[2]. Sleep has a crucial role in many somatic, cognitive, and psychological processes and sleeping well appears to be a health imperative, essential for survival [3]–[6]. However, it is still largely unknown how many exact hours of sleep per day are needed in adults, according to their age, their environmental and socio-cultural characteristics. Some authors recommend 8 hours of sleep whereas others believe 7 hours may be sufficient for adults, with a shorter time being necessary in the elderly [1]–[2], [4], [7]–[9].

Most experts agree that sleep has now to compete more and more with multiple tasks in our today's 24-hour society. It results in a severe sleep duration reduction around the planet, especially for adolescents and young adults [10]–[11]. However, a recent survey in an impressive sample of adults (328,018 subjects) from 10 countries, has shown that reducing sleep time was not a fatality in several countries, except in the most active and young subjects [11]–[12].

Although scientists are still studying the concepts of basal sleep need, increasing evidence tends to show that sleeping too little or too much impacts severely on health with a U-shaped association between short and long sleep duration and morbidity [13]–[25] and even mortality risks [24]–[32] across populations.

The metabolic, behavioral and epidemiological rationale explaining the impact of short sleep duration on health is, however, stronger than that the one of long sleep duration.

Sleep research has certainly shown that sleeping too little can affect memory, immunity, and jeopardize safety [25]–[32]. Chronic short sleep duration (<6 hours) has been associated with an increased risk of obesity, diabetes, hypertension and other cardiovascular diseases [13]–[22]. Acute sleep deprivation (defined as sleeping 25–50% of a normal 8 h night's sleep) contributes to an increased inflammation and disturbs the immunological response [6]–[7], [28], [33]–[34].

The evidence that long sleep is associated with obesity, diabetes, hypertension or other cardiovascular diseases [13]–[18], [20]–[24] and with a 20%–30% higher mortality risk [25]–[27], [29]–[31] has consistently been found even stronger than the associations with short sleep. In the most recent meta-analyses (28–30) the authors suggested that long sleep may be even more detrimental in terms of mortality than short sleep”. However, it is also often advanced that an absence of consensus on the association between long sleep and poor health may be due to methodological biases, confounding, and reverse causation in the interpretation, which may also been advanced for the association with short sleep [31]. An absence of a clear “cut-off” for defining “long sleep”, the heterogeneity of studies with long sleepers, the small numbers of long sleepers studied, the lack of studies in the general population, and the high percentage of older adults in these studies have been underlined [30]–[31]. In older groups, the association between long sleep and mortality may represent an end-of-life process with progressive fatigue and inactivity. Finally, it is also not understood if long sleep may have a health protective impact in short sleepers, when they extended the duration of sleep, as it is suggested by some preliminary studies [35]–[36]. It is however not possible to consider these last results on a public health point of view.

The aim of this study was, therefore, to more precisely study “long sleepers”, using a clear cut-off for sleep duration, in a nationally representative sample of subjects and by so trying to better understand the association between long sleep duration and health.

## Materials and Methods

### Sampling design

Analyses were based on a nationally representative, cross-sectional sample of French adults, collected every 5 years since 1990, the “Baromètre Santé” (BS) (Health Barometer) and conducted by INPES (Institut National de Prévention et d'Education pour la Santé). A common INPES BS study protocol standardizes instrumentation, sampling methods, and data collection procedures at each step, with data cleaning and data set construction performed centrally [37]–[38]. The BS 2010 has been performed between October 2009 and July 2010.

The methodology is a cross-sectional study based on telephone surveys with a randomized selection of households and subjects interviewed, using a computerized system (CATI) to select mobile phone and all home phone numbers with no restriction. If the phone is not answered or busy, the interviewers phone repeatedly for up to 40 times at different times of the week and of the day. These different samples were aggregated and weighted to be representative of the general French population (2008 census [39]), taking into account sex, age groups, address, and size of the agglomeration, academic level, and the number of persons per household. An estimated number of 25000 subjects were found necessary to differentiate significantly groups due to logistic regression on more than 100 factors.

### Subjects

To be eligible, each household had to include at least one French speaking individual between 15 and 85 year-old (yo). The subject was randomly selected from among the household residents; if he/she declined to participate, the household was not selected.

### Ethics

Participation was anonymous and voluntary; the study protocol was approved by the French Commission on Information Technologies and Liberties (Commission Nationale Informatique et Libertés) based on the anonymous nature of the study and the guarantee that the phone numbers selected would be erased from the database after the study.

### Measurements

Sections investigating sleep assessments were introduced for the first time in the BS-2010. These sleep-specific measurements were based on validated sleep-logs recommended for the assessment of sleep in adults [40] on regular (non-leisure) days. As our survey was designed to interview on a single day, we used several items to assess total sleep time (TST) which were:

**- To estimate TST on workdays:**

1) ‘When you have to work (to be active) the next day, at what time do you usually switched off the light to go to sleep?

2) ‘When you have to work (to be active) the next day, at what time do you usually waked up?’

3) ‘How long does it usually take for you to fall asleep?’

4) ‘If you have awakenings during the night, how long do they usually last (minutes)?

TST was defined as the difference between the time at which the participant switches off the light and the time at which they wake up, discounting the time needed to fall asleep + the time awaken.

**- Sleep characteristics and disorders were defined as follows:**

- ***“Long sleep”***: according to the ICSD-3^rd^ edition, “a long sleeper is an adult who typically sleeps 10 hours or more, but feels well and functions without impairment [41].
- ***“Short sleep’***: In adults, sleeping less than 5 to 6 hours during working days is usually considered as the “cut off” for “short sleep” with potential comorbidities [25]–[32]. We then retained sleeping <5 hours to strictly define “short sleepers”.
- ***Insomnia***: according to the DSM-V, ICSD-3, and AASM definitions [41]–[43], insomnia is defined as a: -difficulty initiating sleep (DIS), difficulty maintaining sleep (DMS), early morning awakening (EMA), or non-restorative sleep (NRS) - with a frequency of at least three times per week, for at least one month and with negative impact on daytime functioning.
- ***Hypersomnia, hypersomnolence and other sleep disorders***: Our questionnaire did not allow us to detect with precision subjects with sleep apnea. Regarding hypersomnia, based on ICSD-3 minimum criteria [41] it associates: 1) A complaint of excessive daytime sleepiness occurring regularly or often; 2) a TST>10 hours. We also termed hypersomnolence: sleeping >12 hours on working days [41] and severe hypersomnolence: sleeping >12 hours with a feeling of non-restorative sleep.

### Other variables assessed

- Alcohol use was assessed on AUDIT Score [44] and smoking by questions on daily habits.

- To assess chronic diseases, “the interview included 8 questions on chronic diseases which were carefully designed. The first one was “Do you suffer from any chronic disease, i.e., a disease you had from a long time (at least 6 months) and which may benefit of regular treatment (i.e: diabetis, astma…): Yes, No, Don't know.” Question 2: which one and the interviewer has the possibility of checking the exact (s) chronic disease(s) from an open list of 232 chronic diseases”. Based on these list there are 6 questions on visiting doctors, nurses or other medical staff, hospitalisations in the last 12 months, blood samples or dietetary recommendations, which allow the interviewer to come back to the subject to get more details on the chronic disease. Therefore subjects did not self-report their illnesses but follow specific guidelines which allow us to clearly identify which chronic diseases were diagnosed including psychiatric diseases.

- To complete the psychiatric medical history on the present time, psychological distress was assessed by the SF-36 mental health sub score [45].

- Feelings of precariousness (“i.e. the feeling of having not enough ressources or social support to avoid powerty, professional failure, poor health or life accidents), reports of a serious and traumatic event before 18 yo and verbal, physical or sexual violence during the 12 months prior to the survey were assessed using specific questionnaires on personal background [46].

### Statistical analyzes

Bi-variate and multivariable logistic regression models were applied to investigate whether risk factors were independently associated with short TST and long TST. Analyses were performed using the R 2.12.1 software. The statistics presented (percentages, odds ratios) correspond to weighted and adjusted results. We used Pearson's chi-square tests in the bi-variate analysis; odds ratio (OR) are presented with their 95% confidence interval.

## Results

A total of 24,671 individuals (10,962 males and 13,709 females) participated in the study; those households who refused to participate were replaced by a same profile subject selected in the basis (equally for mobile phones and for home phones). Therefore, the final sample was representative of the French national population (based on the last census) [39]. All individuals completed items on TST and chronic diseases.

### Total sleep time and socio demographics

**Figure 1:**
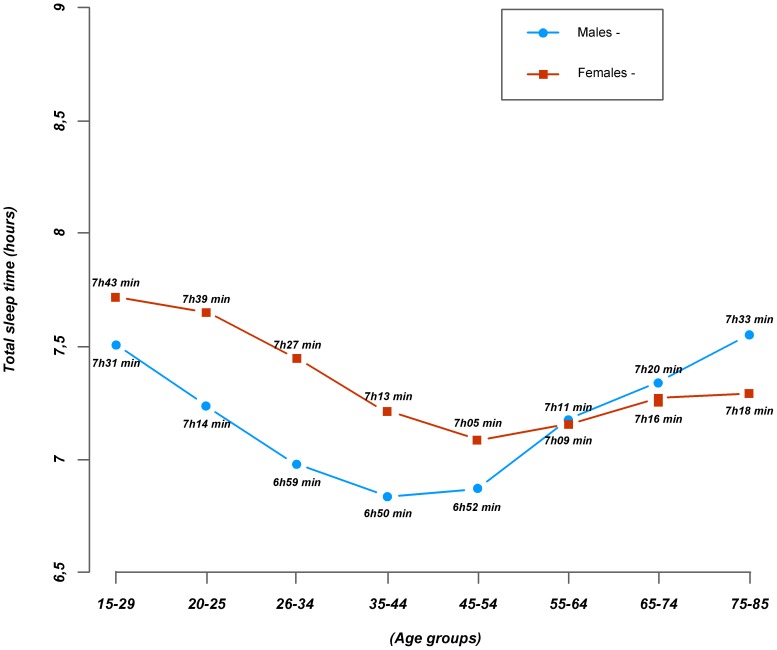
Data taken from the Health Barometer-2010, a nationally representative sample of 24,671 15-85-year-old participants questioned on their sleep duration. Total sleep time was defined as the difference between the time at which the participant switched off the light and the time of day they woke up, discounting the time needed to fall asleep.

The average TST was 7 hours and 13 minutes (+/− 17 minutes) and was significantly longer in females than in males (7 hours 18 minutes (+/− 21 minutes) vs. 7 hours 07 minutes (+/− 22 minutes); p<0,001). TST duration curves with age were similar in males and females (see figure 1), with TST decreasing significantly from 15–19 to 35–44 yo in males and from 15–19 to 45–54 yo in females, before increasing again in the older age groups. Between 55 and 64 yo, there was no difference between males and females for TST, but after 65 yo, males slept longer.

#### Subjects with long or short sleep durations (Tables 1–3)

**Table 1:** Association between TST (Total Sleep Time) and socio-demographic variables.

TST (Total Sleep Time)		<4 hours	[4–5 hours[	[5–6 hours[	[6–7 hours[	[7–8 hours[	[8–9 hours[	[9–10 hours[	> = 10 hours
***Number of individuals***	***24671***	***814***	***1036***	***2516***	***5452***	***7697***	***4860***	***1604***	***692***
***Weighted percentages***	***100***	***3.3***	***4.2***	***10.2***	***22.1***	***31.2***	***19.7***	***6.5***	***2.7***
**Variables**	**N**	**%**	**%**	**%**	**%**	**%**	**%**	**%**	**%**
**Sex ratio**			***	***	***		***	***	*
males (reference)	10962	3.5	5.0	12.2	25.7	31.1	15.5	4.9	2.0
females	13709	3.3	3.7	8.8	20.4	32.4	21.9	7.1	2.5
**Age groups**				*	***	***	***	***	***
15–19 years old (reference)	738	3.3	4.8	9.8	22.0	28.6	16.6	8.3	6.6
20–25 years old	2233	2.8	4.3	8.6	20.1	27.6	21.4	9.1	6.0
26–34 years old	3796	2.7	4.1	9.8	23.0	32.5	19.9	5.7	2.3
35–44 years old	5251	3.6	4.8	11.0	24.6	33.9	16.5	4.4	1.2
45–54 years old	4504	3.4	5.0	11.7	25.9	33.9	14.7	4.3	1.1
55–64 years old	4972	4.1	3.7	10.6	23.1	30.8	19.5	6.4	1.7
65–74 years old	2923	3.3	3.8	10.3	18.5	29.8	24.1	7.8	2.3
75–85 years old	254	4.6	4.9	9.8	15.7	29.4	25.1	8.8	1.7
**Academic level**		***	***	*	***	***	*	***	***
<Baccalaureate (Reference)	11926	4.6	5.4	10.9	21.7	28.5	18.7	7.3	2.9
Baccalaureate	4626	2.0	3.5	10.1	23.6	33.3	20.1	5.4	2.0
> Baccalaureate	8119	1.6	2.5	9.7	25.5	38.5	17.9	3.5	1.0
**Professional category**		***	***	*	***	***	***	***	***
Farmers (Reference)	489	3.9	3.6	9.9	19.3	30.8	23.8	7.3	1.3
Artisans	1369	3.4	5.3	13.0	23.4	29.8	18.4	5.4	1.3
Upper level executive	4660	1.9	2.6	10.6	27.2	36.9	16.5	3.5	0.8
Middle-level	6800	2.1	3.4	9.9	24.4	35.0	19.4	4.5	1.2
White collars	6589	4.1	4.6	9.6	20.3	31.0	20.2	7.2	3.0
Blue collars	4627	4.7	6.2	11.4	22.0	26.6	17.7	7.6	3.7
Other	137	4.7	3.2	7.6	21.9	23.9	11.2	19.5	8.0
**Living alone**		**	***	**		***	***		
No (Reference)	18181	3.2	4.0	10.2	22.9	32.3	19.2	5.9	2.3
Yes	6490	4.3	6.0	11.9	23.0	29.2	16.7	6.6	2.3

Weighted percentages, Pearson's chi square test for bi-variate analysis: ***p<0.001, **p<0.01, *p<0.05.

**Table 2:** Association between TST (Total Sleep Time) and health variables.

TST (Total Sleep Time)		<4 hours	[4–5 hours[	[5–6 hours[	[6–7 hours[	[7–8 hours[	[8–9 hours[	[9–10 hours[	> = 10 hours
***Number of individuals***	***24671***	***814***	***1036***	***2516***	***5452***	***7697***	***4860***	***1604***	***692***
***Weighted percentages***	***100***	***3.3***	***4.2***	***10.2***	***22.1***	***31.2***	***19.7***	***6.5***	***2.7***
**Variables**	**N**	**%**	**%**	**%**	**%**	**%**	**%**	**%**	**%**
**Body Mass Index**		***		***		***		*	**
No overweight, no obesity (Reference)	14894	3.1	4.1	9.6	23.0	32.9	19.1	5.9	2.4
Overweight	7029	3.1	4.6	11.7	23.2	31.2	18.6	5.8	1.7
Obesity	2748	5.6	5.0	11.8	21.7	27.6	17.5	7.6	3.2
**Insomnia**		***	***		***	***		***	***
No (Reference)	20241	3.0	4.0	10.6	23.7	33.4	18.6	5.0	1.7
Yes	4430	4.8	5.7	10.1	20.1	25.9	19.3	9.7	4.5
**Perceived precariousness**		***	***	**		***	***	***	
No perceived precariousness (Reference)	14591	2.4	3.4	9.8	23.1	34.1	19.5	5.6	2.1
At the edge	6619	3.7	4.8	11.2	22.7	30.9	18.7	5.8	2.3
Yes	3461	6.6	7.4	11.7	22.7	24.7	16.1	8.1	2.9
**Violence in the last 12 months**		***	***	***		***	***		
No (Reference)	19961	3.0	3.7	9.9	22.8	32.6	19.4	6.1	2.3
Yes	4710	5.1	7.1	12.7	23.6	27.9	15.8	5.7	2.2
**Serious event before 18 years old**		***	***	**		***	**	*	*
No (Reference)	13156	2.7	3.6	9.8	23.1	33.7	19.5	5.6	2.0
Yes	11515	4.2	5.3	11.1	22.7	29.6	18.0	6.5	2.6
**Psychological distress (SF-36)**		***	***	***		***	***		
No (Reference)	21648	2.8	3.9	10.1	23.1	32.8	19.2	5.9	2.2
Yes	3023	8.0	7.4	12.9	21.7	24.4	15.9	6.9	2.9
**Chronic diseases**		***	**			***			
No (Reference)	18785	3.0	4.1	10.3	23.1	32.4	18.8	5.9	2.3
Yes	5886	4.6	5.2	11.0	22.2	29.5	18.8	6.4	2.2

For each health variable the absence of risk factor was considered as the reference and compared to the other groups within the eight total sleep time groups retained (from <4 hours to > = 10 hours). Weighted percentages expressed the percentages of subject with each risk factors who reported sleeping between x and y hours.

Pearson's chi square test for bi-variate analysis: ***p<0.001, **p<0.01, *p<0.05.

**Table 3:** Multivariate analysis of long sleep and short sleep adjusted odds ratios.

		Short TST<5 hours			Long TST> = 10 hours		
Variables	N	%^a^	Adjusted OR b	CI 95%	% a	Adjusted OR b	CI 95%
**Sex ratio**		***			0.05*		
Males (reference)	10899	8.5	- 1 -		2.0	- 1 -	
Females	13523	7,0	0.8***	[0.7–0.8]	2.5	1.1	[0.9–1.3]
**Age groups**		**0.19**			***		
15–19 years old (reference)	724	8.2	- 1 -		6.6	- 1 -	
20–25 years old	2203	7.1	1.0	[0.7–1.5]	5.8	0.9	[0.6-1.3]
26–34 years old	3756	6.6	0.9	[0.7–1.3]	2.3	0.4***	[0.3–0.6]
35–44 years old	5213	8.3	1.2	[0.9–1.6]	1.2	0.2***	[0.1–0.3]
45–54 years old	4460	8.4	1.2	[0.9–1.6]	1.1	0.2***	[0.1–0.3]
55–64 years old	4932	7.8	1.2	[0.9–1.7]	1.7	0.3***	[0.2–0.4]
65–74 years old	2885	7.1	1.0	[0.7–1.4]	2.3	0.4***	[0.2–0.6]
75–85 years old	249	9.1	1.4	[0.8–2.3]	1.7	0.3*	[0.1–0.8]
**Academic level**		***			***		
<Baccalaureate (Reference)	11776	10.0	- 1 -		2.9	- 1 -	
Baccalaureate	4582	5.5	0.7***	[0.6–0.8]	2.0	0.7**	[0.5–0.9]
> Baccalaureate	8064	4,0	0.5***	[0.4–0.6]	1.0	0.6***	[0.4–0.8]
**Body Mass Index**		***			**		
Normal (Reference)	14846	7.2	- 1 -		2.4	- 1 -	
Overweight	7029	7.8	1.0	[0.9–1.1]	1.7	1.0	[0.8–1.2]
Obesity	2547	10.5	1.2**	[1.1–1.4]	3.2	1.3	[1.0–1.7]
**Professional category**		***			***		
Farmers (Reference)	481	7.6	- 1 -		1.3	- 1 -	
Craftsmen	1363	8.8	1.3	[0.8–1.9]	1.2	1.5	[0.6–3.7]
Upper level executive	4636	4.4	0.9	[0.6–1.3]	0.8	1.1	[0.4–2.6]
Middle-level	6750	5.5	1.0	[0.7–1.4]	1.2	1.3	[0.6–3.1]
White collar	6482	8.7	1.1	[0.8–1.6]	3.0	2.3*	[1.0–5.3]
Blue collar	4578	10.9	1.4	[0.9–2.0]	3.7	2.5*	[1.1–5.7]
Other	132	8.2	1.2	[0.6–2.4]	8.3	3.4*	[1.1–10.3]
**Living alone**		***			**0.96**		
No (Reference)	18012	7.2	- 1 -		2.3	- 1 -	
Yes	6410	10.4	1.4***	[1.2–1.5]	2.3	1.0	[0.8–1.3]
**Insomnia**		***			***		
No (Reference)	20077	7.0	- 1 -		1.7	- 1 -	
Yes	4345	10.4	0.9	[0.8–1.0]	4.5	1.7***	[1.4–2.1]
**Perceived precariousness**		***			**0.18**		
No (Reference)	14475	5.7	- 1 -		2.1	- 1 -	
At the edge	6542	8.3	1.2**	[1.1–1.3]	2.3	0.9	[0.8–1.2]
Yes	3405	14.0	1.7***	[1.5–2.0]	2.9	0.9	[0.7–1.2]
**Violence in the last 12 months**		***			**0.64**		
No (Reference)	19759	6.7	- 1 -		2.3	- 1 -	
Yes	4663	12.2	1.6***	[1.4–1.8]	2.2	0.9	[0.7–1.2]
**Serious event before 18 years old**		***			0.03*		
No (Reference)	13033	6.2	- 1 -		2.0	- 1 -	
Yes	11389	9.4	1.2***	[1.1–1.3]	2.6	1.1	[0.9–1.3]
**Psychological distress (SF-36)**		***			**0.09**		
No (Reference)	21430	6.7	- 1 -		2.2	- 1 -	
Yes	2992	15.3	2.0***	[1.7–2.2]	2.9	1.2	[0.9–1.6]
**Chronic diseases**		***			**0.81**		
No (Reference)	18586	7.1	- 1 -		2.3	- 1 -	
Yes	5836	9.9	1.2*	[1.0–1.3]	2.2	1.3	[1.0–1.6]

a : Weighted percentages. Pearson's chi square test for bivariate analysis: ***p<0.001; **p<0.01; *p<0.05.

b : Adjusted on all shown measures. Wald test: ***p<0.001; **p<0.01; *p<0.05.

- From the all group, 692 (2.7%) individuals reported a TST >10 hours, 143 (0.5%) >11 hours, 43 (0.2%) >12 hours (hypersomnolence).

- Among long sleepers (TST>10 hours), 9.8% (0.26% of the total group) reported an association with being regularly or often sleepy (hypersomnia)**,** vs. 7.8% of the other (non long sleepers) subjects (non-significantly different).

- 0.03% reported severe hypersomnolence (TST>12 hours + non-restorative sleep). Non-restorative sleep was not statistically more reported by “long sleepers” than by the “non long sleepers” group (21.9% vs. 20.8%; NS).

- From the total group, 1,850 individuals reported a TST <5 hours (7.23%) and 814<4 hours (3.3%).

A lower educational level was significantly associated with short or long sleep duration. Long sleep was significantly more frequent in white collar and blue collar workers and short sleep in blue collar workers and craftsmen. Living alone was significantly associated with short sleep.

### Total Sleep Time and health variables (Tables 2 and 3)

#### Weight and obesity

Subjects with long sleep and short sleep duration were significantly more overweight (BMI>25) and obese (BMI>30) than normal sleepers.

#### Insomnia and psychological state

Insomnia was significantly less frequent both in long sleepers and in short sleepers than in the whole group.

Long sleepers did not report more perceived precariousness or violence in the 12 months prior to the survey than did the general population. They, however, did report slightly more psychological distress (SF-36 sub-scale) and violent traumatic events before 18 yo.

Short sleepers reported significantly more psychological distress and more violence in the 12 months prior to the survey, more traumatic events before 18 yo and more perceived precariousness than the whole group.

#### Comorbidities associated with TST (Tables 3 and 4)

Long sleepers did not report more or fewer chronic diseases than did the group as a whole including mental or physical diseases. Conversely, short sleepers claimed significantly more associated chronic diseases; these comorbidities are detailed in Table 4. After multivariable analysis, long sleep was not significantly associated with most of the chronic diseases reported except for a higher rate of mental diseases and a lower rate of ophthalmologic diseases. Conversely, short sleep was significantly associated with most of the comorbidities, including cancer, respiratory diseases, pain and rheumatologic diseases, digestive, hormonal and metabolic diseases, mental diseases, but with less ophthalmologic diseases. Long sleep and short sleep were not significantly associated with a report of an accident in the last 3 months.

**Table 4:** Chronic diseases associated with long sleep or short sleep.

		Short TST<5 hours			Long TST> = 10 hours		
Variables	N	%^a^	Adjusted OR b	CI 95%	% a	Adjusted OR b	CI 95%
**Cardiovascular diseases**							
No (Reference)	22772	7,6	- 1 -		2,3	- 1 -	
Yes	1650	8,8	1,0	[0,9–1,3]	1,9	1,0	[0,7–1,5]
**Cancer**		*					
No (Reference)	24107	7,7	- 1 -		2,3	- 1 -	
Yes	315	12,8	1,5*	[1,0–2,1]	2,7	1,6	[0,8–3,1]
**Respiratory diseases**		**					
No (Reference)	23438	7,6	- 1 -		2,3	- 1 -	
Yes	984	11,4	1,3*	[1,0–1,6]	1,9	0,9	[0,6–1,5]
**Pain and rheumatologic diseases**		***					
No (Reference)	23388	7,5	- 1 -		2,3	- 1 -	
Yes	1034	13,3	1,4**	[1,1–1,7]	1,5	0,8	[0,5–1,3]
**Digestive diseases**		***					
No (Reference)	24049	7,6	- 1 -		2,3	- 1 -	
Yes	373	16,8	1,7***	[1,3–2,3]	1,5	0,7	[0,3–1,7]
**Hormonal, metabolic diseases**		***					
No (Reference)	24049	7,6	- 1 -		2,3	- 1 -	
Yes	373	16,8	1,7***	[1,3–2,3]	1,5	0,7	[0,3–1,7]
**Neurological diseases**							
No (Reference)	24055	7,7	- 1 -		2,3	- 1 -	
Yes	367	10,9	0,9	[0,6–1,3]	2,6	1,7	[0,9–3,1]
**Mental diseases**		**			***		
No (Reference)	24092	7,6	- 1 -		2,2	- 1 -	
Yes	330	14,6	0,9	[0,7–1,3]	8,8	6,0***	[3,9–9,3]
**Urinary Genital diseases**							
No (Reference)	24252	7,7	- 1 -		2,2	- 1 -	
Yes	170	8,1	0,6	[0,3–1,1]	4,1	1,8	[0,7–4,4]
**Dermatological diseases**					**		
No (Reference)	24222	7,7	- 1 -		2,3	- 1 -	
Yes	200	9,5	1,0	[0,6–1,8]	0,5	0,3	[0,0–2,1]
**Ophthalmology**		***					
No (Reference)	24287	7,7	- 1 -		2,2	- 1 -	
Yes	135	2,4	0,4	[0,2–1,0]	7,9	3,3**	[1,6–6,9]
**Accidents in the last 3 months**							
No (Reference)	7712	7,8	- 1 -		2,0	- 1 -	
Yes	353	7,9	1,0	[0,7–1,5]	2,5	0,8	[0,3–1,8]

a : Weighted percentages. Pearson's chi square test for bivariate analysis: ***p<0.001; **p<0.01; *p<0.05.

b : Adjusted on all shown measures. Wald test: ***p<0.001; **p<0.01; *p<0.05.

## Discussion

The aim of the study was to better define and characterize individuals with long sleep durations and, as such, we will limit our discussion to these subjects. We believe that the link between short sleep and health has been well documented; findings that are supported by our data.

- A first strength of our study is that we retrieved data on “long sleepers” from an extensive database of 24,671 individuals from a representative group of the general population. To our knowledge, this is the first study that has observed such a large and representative group of long sleepers: 612 individuals had a TST>10 hours, 143>11 hours and 43>12 hours.

We have here to specify that these long sleepers did not have any other sleep disorders according to the ICSD-3rd international classification [41]: they did not complain of insomnia, non restorative sleep or sleepiness. In a previous study, in which more than 1.1 million subjects (30–102 yo) were observed (American cancer prevention study), Kripke et al. also described a large number of long sleepers: 9541 females and 9600 males reported (based on a single item) sleeping >10 hours per night [26]. However, these participants were older than the general population of USA. In Japan, a prospective survey was also conducted over 12 years with 110,792 subjects: 1663 men and 1269 women reported sleeping >10 hours [47]. These subjects were also older than the general population of Japan (respectively 67.5 and 64.4 yo). Most of the other studies have reported on groups with less than 500 long sleepers, and an overrepresentation of older individuals [13]–[17], [22]–[25], [27], [32].

Our representative sample shows that long sleep is not limited to the elderly, but also concerns a large proportion of young adults (6.6% of 15–19 yo and 6% of 20–25 yo in our survey). Long sleep was very rare in the 35–54 yo class (1.1%) likely because of occupational- and social-related sleep restrictions. As underlined by Grandner et al. [28] in a review on sleep duration and chronic disease: focusing on older individuals may lead to a misinterpretation of the association between long sleep and morbidity. “Finally, mortality risks of long sleep may be associated with general failing health… sleep duration lengthens gradually during the aging process…then poor health would lead to long sleep, rather than long sleep leading to increased mortality.”

In addition to the effects of age, short sleep and long sleep were here significantly associated with a lower educational level, which is often found associated with higher co morbidity rates [14], [17], [20]–[21].

- In contrast to most of the previous studies observing long sleepers [13]–[18], [20]–[25], we did not find a higher risk of comorbidities in long sleepers, except for obesity and psychiatric disorders. These are controversial issues that we will develop further.

Obesity has not been consistently found to be associated with long sleep, despite heterogeneous study designs and populations [13], [15]–[17], [20], [25]–[26]. An analysis of 56,507 subjects, from the US National Health Survey, found an association between long sleep (TST>8 hours) and obesity, diabetes, hypertension and cardiovascular risks [17]. Conversely, the Whitehall II study observed almost no association between long sleep (TST >8 hours) and health variables (including BMI), in a non representative sample of 6,742 subjects from UK and 3,027 from USA [24]. Another large survey of 6461 non-pregnant females did not find any association between long sleep and central obesity after adjustment [14]. In a 10-year prospective survey of 13,269 Japanese, no BMI difference was neither found between long sleepers (>9 hours) and others [13].

The association between long sleep and depression is less controversial. In our study, long sleep was associated with a higher rate of depression, but also a higher report of traumatic events in the past, which may partly explain the depressive complaints. In the Whitehall study, long sleepers (>8 hours) also significantly complained of more depressive symptoms than did normal sleepers [24]. Several other surveys and meta-analyses have also shown a higher rate of depressed patients among long sleepers compared to normal sleepers [13], [32], [48]–[49]. However, after multiple regression analysis, there was no more links between long sleep and depressive symptoms in the Whitehall study [24]. Many other analyses or surveys have neither included depression as a potential confounding factor. We would stress the importance of systematically including an evaluation of depression in future studies in the field. Depression may be associated with dysania (the need to stay in bed without sleeping) and may potentially introduce a bias in the link between long sleep duration and health. We ackowledge that long sleep in subjects with depression may be partially linked to the daily uptake of sedative treatments. However we did not make the choice of reporting all the treatments taken by the patients and did not have the possibility of identifying this potential effect.

Except for obesity and depression, we did not find any significant association between long sleep duration and other comorbidities. This discrepancy between our results and those of previous surveys may be explained by several methodological issues:

- The first was the “cut off” we used to define long sleepers. The heterogeneity of possible cut-offs (>8 h, >8.5 h, >9 h, >10 h) has been identified as a major bias in many reviews and meta-analyses [13], [15], [18], [20]–[21], [24]–[31]. We understand that some authors have used lower “cut-offs” to stratified their samples; however, we do not think that values under 9 h may be valid for representing long sleepers in the general population. In the current study, we used the ICSD-3 definition, for which “a long sleeper is an adult who typically sleeps 10 hours or more, but feels well and functions without impairment”[41].
- A second possible point of confusion stands on how “long sleep” was assessed. Many studies have hypothesized, using just one simple subjective question: “on the average how many hours do you sleep each night” [13]–[14], [16]–[17], [24]–[26] to catch sleep duration. However, recommendations have been made to improve sleep duration estimations, by using sleep logs and different items to calculate TST [42]–[43]. Reviews and meta-analyses have underlined the need for better assessing TST [15], [18], [20]–[21], [24]–[31]. We believe that sleep is such an important risk factor that it should not be assessed by just a single item in this kind of prospective cohort. When, Patel et al used polysomnography and actigraphy to record the sleep patterns of long sleepers (TST>9 hours, self-assessed by sleep logs), they reported that they did spend more time in bed, (+63 minutes) and slept more (+42.8 minutes) than self-reported normal sleepers (p<0.001) (48). These authors found no difference between long sleepers and normal sleepers in terms of the apnea-hypopnea index or the distribution of sleep stages.
- A final point that we have already previously discussed is the age. Most studies have focused on the elderly with or without of elderly potentially associated preexisting comorbidities: cancer, cardiovascular diseases [13]–[17], [22]–[25], [27], [32]. We believe it may introduce a major bias due to the high comorbidities rate in elderly individuals.

Several important meta-analyses and reviews have reported that long sleep is associated with increased mortality [25]–[33]. The association of long sleep with mortality has also been observed in individuals who were healthy at baseline, including a recent study that showed that long sleep in healthy boys was associated with lifetime mortality. Male children who underslept or overslept compared with peers were at increased risk of lifelong all-cause mortality (HR = 1.15, CIs [1.05, 1.27]). Effect sizes were smaller and non significant in females (HR = 1.02, CIs [0.91, 1.14]) [50].

Our study was not prospective and we cannot comment on differences in mortality. However, we agree with the conclusion of the most recent meta-analysis, that “despite a large body of literature, it is premature to conclude. Careful attention must be paid to measurement, response bias, confounding, and reverse causation in the interpretation of associations between sleep duration and mortality [31]”.

Our study has several limitations that restrict the conclusions that can be drawn. Due to the cross-sectional design, it did not allow us to establish causality or temporality. Secondly, information about TST was self-reported by the participants. Nevertheless, self-reported TST assessments have been shown to be valid in long sleepers as registered by both actimetry and polysomnography [48]. Third, comorbidities were assessed based on self reports and were not checked by objective measurements; clinical assessments would have been difficult given the large size of the sample.

## Conclusions

Except for obesity and depression, we found no risk associated with “sleeping too much” in a nationally representative sample of the general population; not surprisingly, “short sleep” was associated with most of these comorbidities.

## References

[1] National Institute of Health. US Department of Health and Human Services. Your guide to healthy sleep. NIH Publication No. 11–5271. (Updated 2011) Accessed 2013 Oct 21. Available: http://www.nhlbi.nih.gov/health/public/sleep/healthy_sleep.pdf?bcsi_scan_43167910db6ab4d9=0&bcsi_scan_filename=healthy_sleep.pdf

[2] World Health Organisation World Regional Office for Europe. European Centre for Environment and Health. WHO technical meeting on sleep and health. (Updated 2005) Accessed 2013 Oct 21. Available: http://www.euro.who.int/__data/assets/pdf_file/0008/114101/E84683.pdf

[3] Diekelmann S, Born J (2010) The memory functions of sleep. Nat Rev Neurosci 11: 114–126. 20046194 10.1038/nrn2762

[4] Siegel JM (2009) Sleep viewed as a state of adaptive inactivity. Nat Rev Neurosci 10: 747–753. 19654581 10.1038/nrn2697 PMC8740608

[5] Dolgin E (2013) Deprivation: a wake-up call. Nature 497: S6–7. 23698507 10.1038/497S6a

[6] Luister FS, Stollo Jr J, Zee PC, Walsh JK (2012) Sleep: A health imperative. Sleep 35: 727–734. 22654183 10.5665/sleep.1846 PMC3353049

[7] Horne J (2011) The end of sleep: ‘sleep debt’ versus biological adaptation of human sleep to waking needs. Biol Psychol 87: 1–14. 20955760 10.1016/j.biopsycho.2010.10.004

[8] Ohayon MM, Carskadon MA, Guilleminault C, Vitiello MV (2004) Meta-analysis of quantitative sleep parameters from childhood to old age in healthy individuals: developing normative sleep values across the human lifespan. Sleep 27: 1255–1273. 15586779 10.1093/sleep/27.7.1255

[9] National Sleep foundation. How Much Sleep Do We Really Need? (Updated 2013) Accessed 2013 Oct 21. Available: http://www.sleepfoundation.org/article/how-sleep-works

[10] Leger D, Beck F, Richard J-B, Godeau E (2012) Total Sleep Time Severely Drops during Adolescence. PLoS One 7(10): e45204. 23082111 10.1371/journal.pone.0045204 PMC3474762

[11] Bin YS, Marshall NS, Glozier N (2012) Secular trends in adult sleep duration: a systematic review. Sleep Med Rev 16: 223–230. 22075214 10.1016/j.smrv.2011.07.003

[12] Bin YS, Marshall NS, Glozier N (2013) Sleeping at the limits: the changing prevalence of short and long sleep durations in 10 countries. Am J Epidemiol 177: 826–833. 23524039 10.1093/aje/kws308

[13] Nagai M, Tomata Y, Watanabe T, Kakizaki M, Tsuji I (2013) Association between sleep duration, weight gain, and obesity for long period. Sleep Medicine 14: 206–210. 23218534 10.1016/j.sleep.2012.09.024

[14] Theorell-Haglöw J, Berglund L, Janson C, Lindberg E (2012) Sleep duration and central obesity in women. Differences between short sleepers and long sleepers. Sleep Medicine 13: 1079–1085. 22841029 10.1016/j.sleep.2012.06.013

[15] Cappuccio FP, D'Elia L, Strazzullo P, Miller MA (2010) Quantity and quality of sleep and incidence of type 2 diabetes: a systematic review and meta-analysis. Diabetes Care 33: 414–420. 19910503 10.2337/dc09-1124 PMC2809295

[16] Chao CY, Wu JS, Yang YC, Shih CC, Wang RH, et al (2011) Sleep duration is a potential risk factor for newly diagnosed type 2 diabetes mellitus. Metabolism Clinical and Experimental 60: 799–804. 20846701 10.1016/j.metabol.2010.07.031

[17] Buxton OM, Marcelli E (2010) Short and long sleep are positively associated with obesity, diabetes, hypertension, and cardiovascular disease among adults in the United States. Soc Sci Med 71: 1027–1036. 20621406 10.1016/j.socscimed.2010.05.041

[18] Guo X, Zheng L, Wang J, Zhang X, Zhang X, et al (2013) Epidemiological evidence for the link between sleep duration and high blood pressure: a systematic review and meta-analysis. Sleep Med 14: 324–332. 23394772 10.1016/j.sleep.2012.12.001

[19] Faraut B, Touchette E, Gamble H, Royant-Parola S, Safar ME, et al (2013) Short sleep duration and increased risk of hypertension: a primary care medicine investigation. J Hypertens 30: 1354–1363. 10.1097/HJH.0b013e32835465e5 22595955

[20] Knutson KL (2010) Sleep duration and cardio metabolic risk: a review of the epidemiologic evidence. Best Pract Res Clin Endocrinol Metab 24: 731–743. 21112022 10.1016/j.beem.2010.07.001 PMC3011978

[21] Capuccio FP, Cooper D, D'Elia L, Strazzullo P, Miller MA (2011) Sleep duration predicts cardiovascular outcomes: a systematic review and meta-analysis of prospective studies. European Heart Journal 32: 1484–1492. 21300732 10.1093/eurheartj/ehr007

[22] Ramos AR, Jin Z, Rundek T, Russo C, Homma S, et al (2013) Relation between Long Sleep and Left Ventricular Mass (from a Multiethnic Elderly Cohort). Am J Cardiol 112: 599–603. 23711813 10.1016/j.amjcard.2013.04.029 PMC3770129

[23] Ramos AR, Dong C, Elkind MS, Boden-Albala B, Sacco RL, et al (2013) Association between sleep duration and the Mini-Mental Score: The Northern Manhattan Study. J Clin Sleep Med 9: 669–673. 23853560 10.5664/jcsm.2834 PMC3671331

[24] Stranges S, Dorn JM, Shipley MJ, Kandala NB, Trevisan M, et al (2008) Correlates of short and long sleep duration: A cross-cultural comparison between the United Kingdom and the United States. The Whitehall II study and the Western New York Health Study. Am J Epidemiol 168: 1353–1364. 18945686 10.1093/aje/kwn337 PMC2727192

[25] Kronholm E, Laatikainen T, Peltonen M, Sippola R, Partonen T (2013) Self-reported sleep duration, all-cause mortality, cardiovascular mortality and morbidity in Finland. Sleep Medicine 12: 215–221. 10.1016/j.sleep.2010.07.021 21317033

[26] Kripke DF, Garfinkel L, Wingard DL, Klauber MR, Marler MR (2002) Mortality associated with sleep duration and insomnia. Arch Gen Psychiatry59: 131–136. 10.1001/archpsyc.59.2.131 11825133

[27] Maia Q, Grandner MA, Findley J, Gurubhagavatula I (2013) Short and long sleep duration and risk of drowsy driving and the role of subjective sleep insufficiency. Accid Anal Prev (59) 618–622. 10.1016/j.aap.2013.07.028 PMC3771664 23973762

[28] Grandner MA, Hale L, Moore M, Patel NP (2010) Mortality associated with short sleep duration. The evidence, the possible mechanisms, and the future. Sleep Med Rev 14: 191–203. 19932976 10.1016/j.smrv.2009.07.006 PMC2856739

[29] Galicchio L, Kalesan B (2009) Sleep duration and mortality: a systematic review and metaanalysis. J. Sleep Res 18: 148–158. 19645960 10.1111/j.1365-2869.2008.00732.x

[30] Cappuccio FP, D'Elia L, Strazzullo P, Miller MA (2010) Sleep duration and all-cause mortality: a systematic review and meta-analysis of prospective studies. Sleep 33: 585–592. 20469800 10.1093/sleep/33.5.585 PMC2864873

[31] Kurina LM, McClintock MK, Chen JH, Waite LJ, Thisted RA, et al (2013) Sleep duration and all-cause mortality: a critical review of measurement and associations. Annals of Epidemiology 23: 361–370. 23622956 10.1016/j.annepidem.2013.03.015 PMC3660511

[32] Cohen-Mansfield J, Perach R (2012) Sleep duration, nap habits, and mortality in older persons. Sleep 35: 1003–1009. 22754047 10.5665/sleep.1970 PMC3369219

[33] Ackermann K, Revell VL, Lao O, Rombouts EJ, Skene DJ, et al (2012) Diurnal rhythms in blood cell populations and the effect of acute sleep deprivation in healthy young men. Sleep 35: 933–940. 22754039 10.5665/sleep.1954 PMC3369228

[34] Faraut B, Boudjeltia KZ, Vanhamme L, Kerkhofs M (2012) Immune, inflammatory and cardiovascular consequences of sleep restriction and recovery. Sleep Med Rev 16: 137–149. 21835655 10.1016/j.smrv.2011.05.001

[35] Markwald RR, Melanson EL, Smith MR, Higgins J, Perreault L, et al (2013) Impact of insufficient sleep on total daily energy expenditure, food intake, and weight gain. Proc Natl Acad Sci 110: 5695–5700. 23479616 10.1073/pnas.1216951110 PMC3619301

[36] Gumenyuk V, Korzyukov O, Roth T, Bowyer SM, Drake CL (2013) Sleep extension normalizes ERP of waking auditory sensory gating in healthy habitually short sleeping individuals. PLoS One 8(3): e59007. 23520548 10.1371/journal.pone.0059007 PMC3592823

[37] Beck F, Guilbert P, Gautier A (2007) Baromètre santé 2005, attitudes et comportements de santé. Saint-Denis, France. INPES Ed; p 608.

[38] Beck F, Gautier A, Guignard R, Richard JB (2011) Une méthode de prise en compte du dégroupage total dans le plan de sondage des enquêtes téléphoniques auprès des ménages. In: Tremblay ME, Lavallée P, El Hadj Tirari M. Pratiques et Méthodes de sondage. Paris: Dunod, Collection Sciences Sup; p 310–14.

[39] Institut National de Statistiques et des études économiques (INSEE) (2010) Démographie en France 2010. (Updated 2011; cited 2013 October 21). Available: http://www.insee.fr.

[40] Morgenthaler TI, Lee-Chiong T, Alessi C, Friedman L, Aurora RN, et al (2007) Practice parameters for the clinical evaluation and treatment of circadian rhythm sleep disorders. An American Academy of Sleep Medicine report. Standards of Practice Committee of the American Academy of Sleep Medicine. Sleep 30: 1445–1459. 18041479 10.1093/sleep/30.11.1445 PMC2082098

[41] American Academy of Sleep Medicine (2014) International Classification of Sleep Disorders, 3rd ed: Diagnostic and Coding Manual. Darien, Ill: American Academy of Sleep Medicine.

[42] American Psychiatric Association (2013) Diagnostic and statistical manual of mental disorders, Fifth Edition, (DSM-V). American Psychiatric Publishing. Washington DC USA and London England.

[43] Edinger JD, Bonnet MH, Bootzin RR, Doghramji K, Dorsey CM, et al (2004) American Academy of Sleep Medicine Work Group. Derivation of research diagnostic criteria for insomnia: report of an American Academy of Sleep Medicine Work Group. Sleep 27: 1567–1596. 15683149 10.1093/sleep/27.8.1567

[44] Saunders JB, Aasland OG, Babor TF, de la FuenteJR, Grant M (1993) Development of the Alcohol Use Disorder Identification Test (AUDIT): WHO collaborative project on early detection of persons with harmufl alcohol consumption-II. Addiction 88: 791–804. 8329970 10.1111/j.1360-0443.1993.tb02093.x

[45] Leplège A, Ecosse E, Verdier A, Perneger T (1998) The French SF-36 Health Survey: translation, cultural adaptation and preliminary psychometric evaluation. Journal of clinical epidemiology 51: 1013–1023. 9817119 10.1016/s0895-4356(98)00093-6

[46] Legleye S, Beck F, Peretti-Watel P, Chau N, Firdion JM (2010) Suicidal ideation among young French adults: association with occupation, family, sexual activity, personal background and drug use. J Affect Disord 23: 108–115. 10.1016/j.jad.2009.10.016 19892406

[47] Ikehara S, Iso H, Date C, Kikuchi S, Watanabe Y, et al (2009) Association of sleep duration with mortality from cardiovascular disease and other causes for Japanese men and women: the jaccstudy. Sleep 32: 259–301. 10.1093/sleep/32.3.295 PMC2647783 19294949

[48] Patel SR, Blackwell T, Ancoli-Israël S, Stone KS (2012) Sleep characteristics of self reported long sleepers. Sleep 35: 641–648. 22547890 10.5665/sleep.1822 PMC3321423

[49] Stenholm S, Kronholm E, Bandinelli S, Guralnik JM, Ferrucci L (2011) Self-reported duration and time in bed as predictors of physical function decline: results from the InChianti study. Sleep 34: 1583–1593. 22043129 10.5665/sleep.1402 PMC3198213

[50] Duggan KA, Reynolds CA, Kern ML, Friedman HS (2014) Childhood Sleep Duration and Lifelong Mortality Risk. Health Psychol. Mar 3. 10.1037/hea0000078 PMC4468018 24588628

